# Local cooling and warming effects of forests based on satellite observations

**DOI:** 10.1038/ncomms7603

**Published:** 2015-03-31

**Authors:** Yan Li, Maosheng Zhao, Safa Motesharrei, Qiaozhen Mu, Eugenia Kalnay, Shuangcheng Li

**Affiliations:** 1 College of Urban and Environmental Sciences, Peking University, Beijing 100871, China; 2 Department of Atmospheric and Oceanic Science, University of Maryland, College Park, Maryland 20742, USA; 3 Key Laboratory for Earth Surface Processes of The Ministry of Education, Peking University, Beijing 100871, China; 4 The Institute for Physical Science and Technology, University of Maryland, College Park, Maryland 20742, USA; 5 Department of Geographical Sciences, University of Maryland, College Park, Maryland 20742, USA; 6 Department of Physics, University of Maryland, College Park, Maryland, 20742, USA; 7 National Socio-Environmental Synthesis Center (SESYNC), Annapolis, Maryland 21401, USA; 8 Numerical Terradynamic Simulation Group, Department of Ecosystem and Conservation Sciences, University of Montana, Missoula, Montana 59812, USA

## Abstract

The biophysical effects of forests on climate have been extensively studied with climate models. However, models cannot accurately reproduce local climate effects due to their coarse spatial resolution and uncertainties, and field observations are valuable but often insufficient due to their limited coverage. Here we present new evidence acquired from global satellite data to analyse the biophysical effects of forests on local climate. Results show that tropical forests have a strong cooling effect throughout the year; temperate forests show moderate cooling in summer and moderate warming in winter with net cooling annually; and boreal forests have strong warming in winter and moderate cooling in summer with net warming annually. The spatiotemporal cooling or warming effects are mainly driven by the two competing biophysical effects, evapotranspiration and albedo, which in turn are strongly influenced by rainfall and snow. Implications of our satellite-based study could be useful for informing local forestry policies.

## Abstract (web summary)

Local climatic effects of forests remain poorly understood due to the coarse spatial resolution of models and field observations. Here, the authors use global satellite data to analyse the spatiotemporal cooling or warming effects of tropical, temperate and boreal forests on climate.

## Graphical abstract

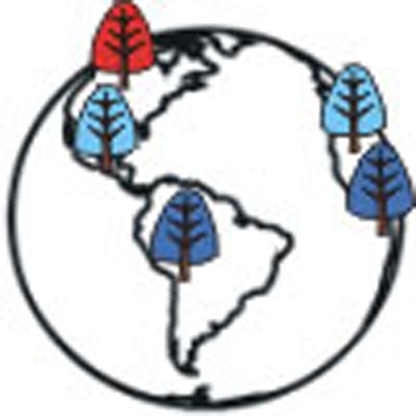

Global forests experienced dramatic changes in the 21st century, with increased deforestation rates in the tropics and decreased rates in the other regions, both largely driven by anthropogenic land use practices^1^ and various natural causes (for example, forest fires^2^ and extreme climate-induced tree mortality^3^). These changes are likely to affect the climate via both biogeochemical^4^ and biophysical processes^5^ and contribute to climate change. Biogeochemical effects (for example, changes in carbon sinks) of forestry can alter atmospheric CO_2_ concentration through changing tree cover. Biophysical effects (for example, albedo and evapotranspiration (ET))^6^ are much more complicated due to high spatial heterogeneity and can cause either warming or cooling^7^,^8^. Therefore, carbon sequestration benefits of forests on climate can be either enhanced or counteracted by biophysical mechanisms^9^,^10^. The net climate benefit could be marginal or even negative when considering forests as a mitigation strategy^7^,^11^. This gives rise to an ongoing debate on the role of extratropical forests on climate as most climate model results indicate a biophysical warming^5^, making afforestation and reforestation at mid latitudes a less favourable practice for mitigation. In contrast, more recent empirical studies tend to show the opposite^12^,^13^,^14^.

Although forests have dominant biophysical impacts at the local scale, which are more relevant for management practices^13^, research progress on the abovementioned question (and debate) is hindered by certain limitations of existing methodology. In modelling approaches, global climate models still cannot reproduce local climate effects reliably due to their coarse spatial resolution and uncertainties in physical processes, parameterization, and input data^15^. Regional climate models can improve spatial resolution and physical representation at the local and regional levels, but global simulation with regional climate models is not yet feasible due to computational limitations^16^. For observational approach, *in situ* measurements (for example, Fluxnet, field experiments, and weather stations) can offer new insights^17^,^18^ and provide local evidence to verify model results. But in most cases, field data are collected from geographically restricted areas, inadequate to address global forests with high spatial variability in biophysical characteristics and climate conditions. Because of the mismatch in spatial scale between the observed and simulated climate signals, it is still unclear to what extent the observed local-scale climatic and biophysical effects could be extrapolated over larger areas and serve as a reference to model results.

Repeated and consistent observations from satellites allow us to investigate the local effects of forests from a global perspective at high resolution and can improve our understanding of the forestry policy outcomes. In this study, we use satellite data from the moderate resolution imaging spectroradiometer (MODIS) to investigate the biophysical impact of forests on local temperature at a global coverage and explore the controlling mechanisms. We compare land surface temperature (LST), ET, albedo, and other surface energy components between forest and nearby open land (grassland and cropland) from more than 11,000 samples across the globe (Supplementary Fig. 1). Open land is a proxy for non-forest land, representing either a consequence of deforestation or suitable land for afforestation/reforestation in the future. The impact of forests on local temperature can be expressed as the LST difference (ΔLST) of forest minus open land:


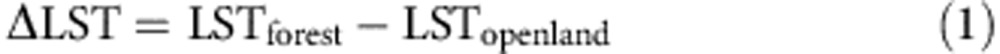


Positive (negative) ΔLST indicates a warming (cooling) effect of forests. Differences in albedo (Δalbedo), ET (ΔET), and other variables are defined similarly.

Our results show that the effects of forests on local temperature have distinctive latitudinal patterns—ranging from strong cooling in the tropics to moderate cooling in the temperate regions and to warming in the high latitudes. The temperature effects also have diurnal asymmetric features, mostly daytime cooling and nighttime warming, and exhibit considerable spatial and seasonal variations. The observed patterns are largely controlled by the biophysical mechanisms (albedo and ET) as well as the background climate (rainfall, snow, and shortwave radiation) through the surface energy balance.

## Results

### Diurnal effects of forest on ΔLST and their spatial variations

**Figure 1:**
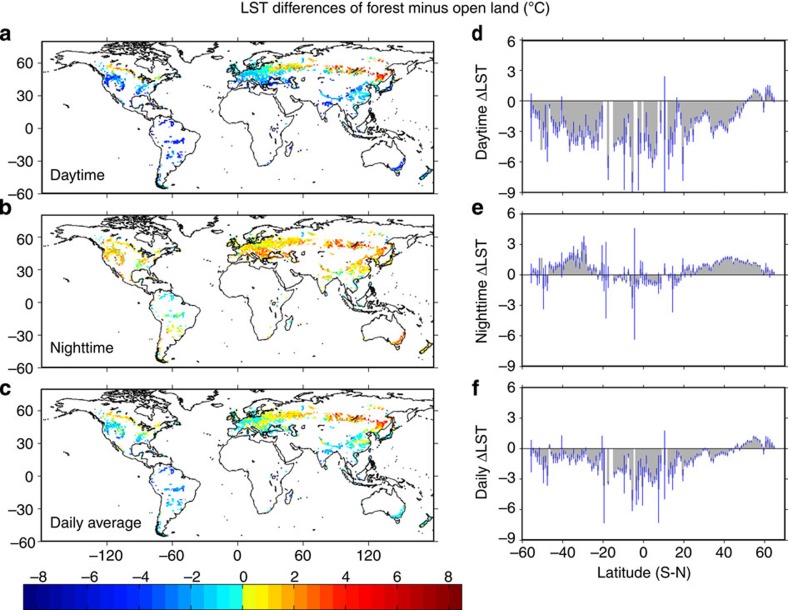
Annual LST difference of forest minus open land. Effect of forest on temperature is represented by ΔLST (forest minus open land), where positive (negative) values indicate a warming (cooling) effect of forests. (**a**–**c**) Spatial pattern (averaged on 1 × 1° grids) and (**d**–**f**) corresponding latitudinal dependence of ΔLST for daytime, nighttime, and daily averages (blue line denotes 95% confidence interval (CI) estimated by *t*-test). Latitude bars with CI out of display range are not drawn. Latitude statistics are in Supplementary Table 1.

During daytime, cooling is the dominant effect in the majority of forests relative to open land. There is more cooling near the equator and less near the poles (Fig. 1a,d), resulting in the largest cooling effect in the tropics (20°S–20°N), moderate cooling in the mid latitudes (20°N–50°N) and warming in boreal forests (50°N–90°N). The latitude pattern is not perfectly symmetrical between the northern and southern hemispheres. Southern temperate forests (20°S–50°S) have slightly larger cooling than northern and there is no warming in the southern high-latitude forests (50°S–90°S).

During night, most forests are warmer than open land, but the magnitude of night warming is generally lower than that of the daytime cooling effect. The latitudinal pattern of ΔLST at night is also different from that of daytime (Fig. 1b,e). Tropical forests exhibit almost no night warming but have a slight cooling effect. The greatest night warming occurs in the mid latitudes (20°S–50°S and 20°N–50°N) in both hemispheres. Night warming in high-latitude forests is much greater in the northern hemisphere (NH) than in the southern hemisphere (SH).

Daily average effects are largely determined by the daytime ΔLST because of its greater magnitude than nighttime (Fig. 1c,f), whereas the nighttime effect can enhance, counteract, or even reverse the daytime effect. Tropical forests show strong daily cooling of −2.41±0.10 °C because of the consistent cooling during both day and night. Here the confidence interval is estimated by the *t*-test at 95% confidence level for a given geographic range or time period to examine whether the difference in the mean values of forest and open land is significant (same for other variables). However, it does not reflect uncertainties from the satellite data, the window searching method, and interannual variability. In the mid latitudes, daytime cooling is largely offset by night warming, leading to a weak daily cooling of −0.27±0.03 °C in NH and a larger cooling of −0.97±0.07 °C in SH. Due to consistent diurnal warming signals, boreal forests show a strong daily warming effect of 0.79±0.03 °C. A small daily cooling of −0.50±0.19 °C is observed for the southern high-latitude forests mainly due to insignificant night warming. The transitional latitude that separates cooling and warming is around 45°N (Fig. 1f). This latitudinal pattern that we identified through LST had also been observed in the air temperature difference between forest and open land^18^,^19^, although these studies found a different transitional latitude of 35°N.

### Seasonal effects of forests on ΔLST and latitudinal patterns

**Figure 2:**
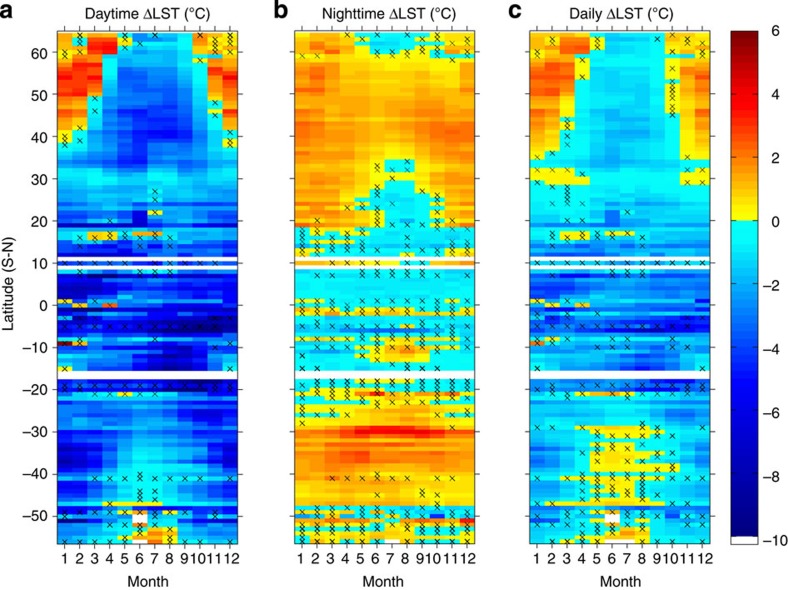
Seasonal and latitudinal variations of LST differences of forest minus open land. (**a**) Daytime ΔLST. (**b**) Nighttime ΔLST. (**c**) Daily average ΔLST. Grids with crosses indicate that the differences are insignificant at 95% by *t*-test. Latitude statistics are given in Supplementary Table 2.

Tropical forests maintain a strong year-round cooling effect, while a clear seasonal variation can be seen in mid- and high-latitude forests (Fig. 2, Supplementary Table 2). In the warm season (mostly growing season), daytime cooling dominates all forests and peaks in magnitude, which, together with weaker night warming, results in a strong net daily cooling effect. In the cold season (dormant season), daytime warming spreads to most mid- and high-latitude forests and, combined with widespread night warming, leads to a maximum daily warming effect. This seasonal variation shifts the transitional latitude, which separates cooling and warming, in both hemispheres. The transitional latitude moves poleward; it disappears in the warm season and extends to around 30° in the cold season (Fig. 2c).

### Biophysical controls on effects of forests on ΔLST

**Figure 3:**
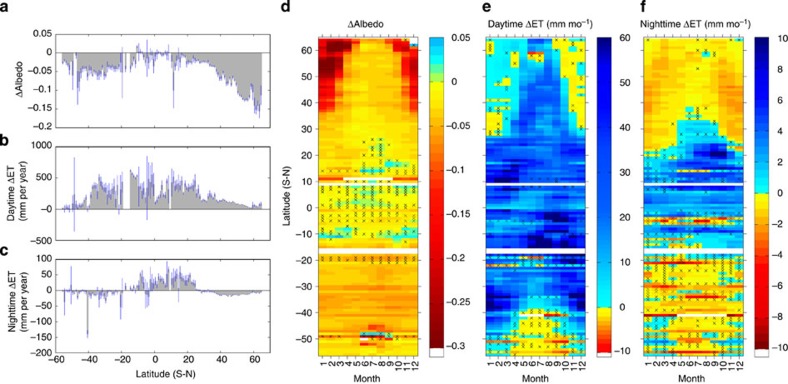
Albedo and ET differences of forest minus open land. Seasonal and latitudinal variations of Δalbedo (**a**,**d**), daytime ΔET, (**b**,**e**) and nighttime ΔET (**c**,**f**). Negative Δalbedo shows that forest has lower albedo than open land. Positive ΔET shows that forest has higher ET than open land. Latitude bars with CI out of display range are not drawn. See latitude statistics in Supplementary Tables 3 and 4.

Figure 3 shows that Δalbedo and ΔET are the two major biophysical factors underlying latitudinal (Fig. 1) and seasonal (Fig. 2) variations of ΔLST. Forests generally have lower albedo than open land (negative Δalbedo in Fig. 3a). Note that albedo difference (Δalbedo) in the tropics is much smaller (−0.02±0.002, Supplementary Table 3) than the previous *in situ* measurements^20^,^21^. An alternative MODIS-derived albedo data set, GLASS (Global LAnd Surface Satellites), yields no improvement for this underestimation issue (Supplementary Fig. 2). This could probably reflect MODIS underestimation of albedo for crops and grass^22^.

With lower albedo, forests absorb more shortwave radiation during daytime (positive shortwave anomalies ΔSW in Supplementary Fig. 3a), potentially leading to a warming effect. However, this net energy gain is offset by a greater latent heat loss via higher ET in forests (positive ΔET, Fig. 3b), resulting in a cooling effect. The strength of albedo warming generally increases with latitude, while the strength of ET cooling decreases (their net effect, ΔSW-ΔET, can be seen in Supplementary Fig. 3b). Across latitudes, the strongest cooling is in tropical forests where high ET cooling completely offsets albedo warming. In temperate forests, ET cooling is lower and albedo warming is higher compared with the tropics, resulting in moderate cooling (stronger in SH due to larger ΔET). In boreal forests, albedo warming completely surpasses the negligible ET cooling, causing pronounced warming. Absence of warming in high latitudes of SH (Fig. 1a,d) is a result of a much weaker albedo effect due to less snow presence than the corresponding areas in NH. On a seasonal scale, for extratropical forests with strong seasonality, significant cooling in the warm season (Fig. 2a) is the net effect of strong ET cooling and weak albedo warming (Fig. 3d,e). In contrast, substantial warming in the cold season (Fig. 2a) is a consequence of strong albedo warming combined with weak ET cooling (Fig. 3d,e). It is interesting to note that tropical forests (in both hemispheres) with little seasonality have a stronger cooling effect (Fig. 2a) and the largest ΔET (Fig. 3e) during the dry season. Observational evidence of the Amazon^21^ found a sharp decline of grass ET during the dry period, whereas trees have higher ET^23^, thus a larger ET difference. The contrasting response is because forest trees with deeper roots have higher ET efficiency than grass and can maintain a large uptake of soil water, whereas grass is more strongly affected by water limitation in the upper soil layer^21^,^24^.

The absence of night warming in tropical forests and the presence of strong night warming in temperate forests (Fig. 1e) are related to shortwave energy absorption during daytime (ΔSW). Nighttime warming in forests is primarily a result of releasing heat energy stored during daytime^25^,^26^. Larger heat capacity allows forests to lose heat more slowly and canopies to stay warm at night^27^. The more energy forests receive during daytime, the stronger the night warming. This is supported by the positive relationship between ΔSW and nighttime ΔLST (*R*=0.30, *P*<0.001) as well as good correspondence between their latitudinal patterns (Fig. 1e and Supplementary Fig. 3a). In addition to the energy legacy from daytime, nighttime ET may also contribute to the nighttime temperature effect. There is a negative relationship between nighttime ΔET and nighttime ΔLST (*R*=−0.29, *P*<0.001), indicating the ET cooling effect still exists at night. This is particularly important for tropical forests because the amount of nighttime ET surplus in the tropics is comparable to the daytime surplus in some high latitudes (Fig. 3c). Thus, the weak warming effect induced by ΔSW can be further suppressed by strong nighttime ET cooling, leading to slight night cooling for tropical forests (Fig. 1e). By contrast, in extratropics, a slightly negative ΔET (probably due to a drier climate) could strengthen the night warming caused by the release of the stored daytime energy (ΔSW), especially in the mid latitudes (Fig. 3c). This hypothesis is further supported by the similar seasonal patterns exhibited by negative nighttime ΔET and positive nighttime ΔLST (Figs 3f and 2b).

### Impact of climate conditions on biophysical effects and ΔLST

**Figure 4:**
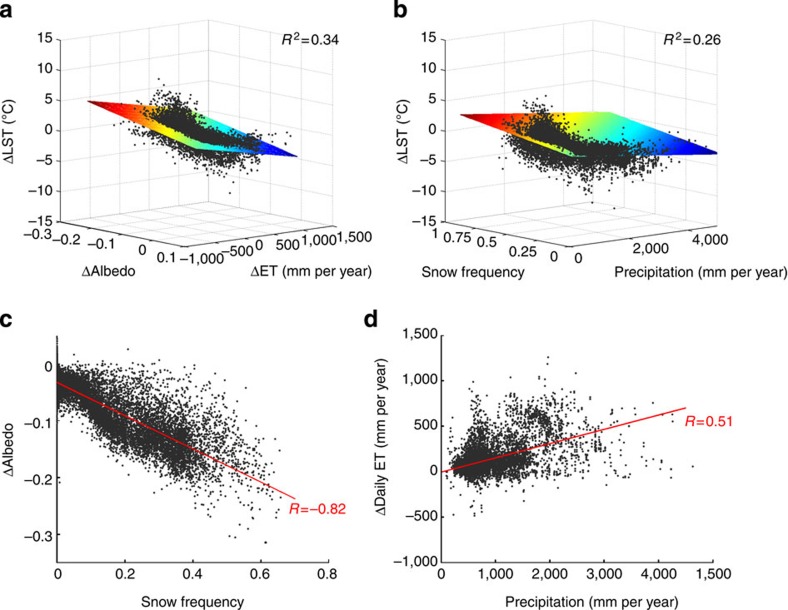
Impacts of biophysical and climate variables on the LST difference of forest minus open land. (**a**) Effects of Δalbedo and ΔET on daily ΔLST. (**b**) Effects of rainfall and snow frequency on daily ΔLST. The regression surfaces in **a** and **b** are computed by the least squares method. (**c**) Relationship between snow frequency and Δalbedo (*R*=−0.82). (**d**) Relationship between precipitation and ΔET (*R*=0.51). Black dots in (**c**,**d**) are data samples of Δalbedo or ΔET and red solid lines are the best fit lines.

ΔAlbedo and ΔET together explain 34% of the spatial variance in annual daily ΔLST (Fig. 4a). Background climate^28^—in particular, form of precipitation, that is, snow versus rainfall—can also influence daily ΔLST (Fig. 4b, *R*^2^=0.26) through its effects on Δalbedo and ΔET. ΔAlbedo is highly negatively correlated with snow frequency (*R*=−0.82, *P*<0.001, Fig. 4c). This is expected because of the larger impact of snow on albedo in open land than in forest, which in turn is caused by the snow masking effect on grass^29^. It also suggests the presence of snow can significantly amplify the albedo warming effect, as in boreal forests (Fig. 1). Using annual precipitation as an index for water availability, the positive correlation between daily ΔET and precipitation (*R*=0.51, *P*<0.001, Fig. 4d) shows not only higher ET efficiency of trees than grass^24^ but also the influence of moisture conditions^30^. Higher precipitation reduces the likelihood of moisture stress and facilitates trees to remove the absorbed shortwave energy in the form of latent heat^28^,^31^, leading to greater ΔET in these regions. Moreover, background shortwave radiation plays a role in ΔET too (*R*=0.56, *P*<0.001) because the available energy becomes more important when moisture is not a limiting factor for ET^30^ (for example, in the tropics). In general, the ET cooling effect in forests can be enhanced under humid conditions and constrained under dry conditions, suggesting a more likely cooling for the presence of forests in wet regions or warming in dry regions, mechanisms supported by both empirical^19^ and modelling^28^,^31^ studies. It also explains why temperate forests in SH have a stronger cooling effect than in NH even though they have very close Δalbedo (Supplementary Table 3), a result of larger ΔET in SH due to more rainfall and shortwave radiation (Supplementary Table 4).

These facts highlight the important role of background climate in determining the general patterns of biophysical variables and, consequently, the impact of forests on temperature. Future climate change (for example, in snow and rainfall) is likely to influence these observed patterns^28^.

## Discussion

While climate models cannot reliably reproduce local effects and field data have insufficient coverage, our analysis of satellite data at global scale provides new evidence to fill the knowledge gap in the local-scale temperature effects of forests. We found that forests affect local temperature through biophysical mechanisms (albedo and ET), consistent with the existing mechanisms in the models. Since general spatial patterns from model simulations show a close resemblance to our results for most forests except in the mid latitudes, we can use observed local-scale biophysical and climatic effects from satellite data to infer large-scale impacts. The effect of temperate forest on temperature is less clear^5^ as can be seen from the mixed results of the models. Nevertheless, most of them indicate a biophysical warming due to the albedo effect^32^. Inconsistencies between models could arise from model uncertainties such as calculating ET^33^,^34^ and soil moisture^31^, or from important atmospheric^6^ and oceanic^24^ feedbacks that can greatly influence or even override the observed patterns here. Past evidence^6^ shows that temperate forests could have a cooling (warming) effect without (with) atmospheric feedback. Although most of the ongoing land use changes, such as forest management, are occurring locally and are presumably too small to cause such feedback^18^, it is not yet well known at which scale these feedbacks can be triggered and become dominant^33^,^35^,^36^.

The biophysical effects of forests that we present here show global patterns that are consistent with other observational analyses^13^,^37^. However, we find a different transitional latitude compared with previous studies using air temperature^18^,^19^. This leads to a discrepancy for temperate forests between 35°N–45°N, where air temperature indicates warming but our analysis and other empirical studies based on LST indicate cooling^12^,^14^,^38^. It should be emphasized that despite the inconsistent sign, the absolute magnitude of temperature difference is rather small. Such inconsistency could be caused by (i) inherent differences between LST and air temperature^39^; (ii) LST retrieval from clear-sky conditions^40^; and (iii) the fact that day and night LST from Aqua satellite approaches the daily maximum and minimum, thus temperature effects based on LST may not suggest the same effects based on air temperature. These can change the relative importance of daytime cooling and nighttime warming and thus the sign of the daily temperature effect. Specifically, between 35°N and 45°N, ΔLST shows stronger daytime cooling than night warming, whereas air temperature difference shows stronger night warming than daytime cooling^19^. We further investigate this issue by examining the latitudinal difference between LST and air temperature at weather stations that are classified as forest and open land (see ‘LST versus air temperature’ in Methods). Results support a potential shift of transitional latitude with LST and suggest a smaller air temperature difference between forest and open land than that derived from LST along with a smaller LST difference under all sky conditions than under clear-sky only. Regarding the effect of forests on local temperature, quantitative evidence from ΔLST may suggest an upper limit for air temperature change in response to land cover change^41^.

Mixed results from both modelling and observations tend to converge, revealing the transitional nature of temperate forests^19^,^42^. Forests north of 45°N tend to be warming, like boreal forests, and those south of 35°N tend to be cooling, like tropical forests. Between 35°N–45°N, it is possible that different biophysical effects for forests (ET versus albedo) within this range exhibit comparable strengths (Supplementary Fig. 3b), making their net effects weak and more susceptible to other factors (for example, land–atmosphere interaction and different temperature metrics) that can alter the relative role of the biophysical effects. By contrast, tropical and boreal forests are clearly divergent in the biophysical effects due to the dominant ET and albedo effects, respectively, as is known from the literature^5^.

In practice, biophysical effects can be influenced by background climate (for example, snow, solar shortwave radiation, and rainfall) and, more importantly, by various human management practices^43^ in forests, grasslands, and croplands (for example, irrigation of croplands during the growing season that has a cooling effect^44^,^45^). A recent study showed that the biophysical impact of land management, such as intensification, can influence surface climate as much as land cover change, even without a change in the land cover type^43^. Given the complex processes occurring at local scale, the effect of forests is expected to have high spatial variability even within the same climate zone. Therefore, a clear understanding of the climatic impacts of forests in the context of land cover change requires an understanding of the specific biophysical changes induced by human activity superimposed on climate condition.

The comprehensive, global view of the impact of forests on local temperature provided in our study can improve the general understanding of the effects of current forestry activities, such as afforestation^25^ or deforestation^18^, on local climate. This understanding is instrumental for land use management and planning.

## Methods

### Data and analysis

MODIS 8-day Aqua LST data (from MYD11C2) are retrieved in clear-sky conditions^40^ and have overpass time at 1:30 and 13:30 hours, which are close to the times of daily minimum and maximum temperature. We extract LST with estimated emissivity error <=0.02 and LST error <=1 K. It should be noted that the Aqua LST data start from July 2002, while other MODIS data incorporate information from the Terra satellite and start from January 2002.

The 16-day MODIS shortwave albedo product (MCD43C3) comprises black-sky and white-sky albedo. Calculation of the actual (blue-sky) albedo, which is a combination of black-sky and white-sky albedo, requires the ratio of the direct to diffusive shortwave radiation. We simply assume the blue-sky albedo to be the average of black-sky and white-sky albedo because their difference is very small and they are highly correlated (*R*=0.9992). Moreover, there is no consensus on the choice of albedo in the literature (for example, Luyssaert *et al.*^43^ and Peng *et al.*^25^ used black and white albedos, respectively). In fact, the choice of specific albedo (white, black, or blue sky) would have little impact on our analysis. Regarding data quality, only data with a QC flag of 0 (best quality), 1 (good quality), or 2 (mixed quality) are used. Snow information is included as a part of the albedo data set. We calculate snow frequency as the percentage of days in a year where the land surface is covered by snow, reflecting the duration of snow cover.

In addition, we use another MODIS based 8-day albedo product, GLASS^46^,^47^, from 2002 to 2012. The selected QC flags are ‘00’ and ‘01’, indicating uncertainty of <5 and 10%, respectively. The GLASS albedo data are used to verify the latitudinal pattern of Δalbedo in Fig. 3a.

Monthly ET is estimated by an algorithm^48^ that calculates daytime and nighttime ET and other variables including latent heat and shortwave radiation.

Land cover data from MCD12C1 with IGBP classification in 2012 are used to define forest and open land. Five forest types (evergreen needleleaf, evergreen broadleaf, deciduous needleleaf, deciduous broadleaf, and mixed forests) are combined into a single ‘forest’ category. ‘Open land’ refers to grassland and cropland. Only forest and open land (grass/crop) pixels with over 80% covered area are taken into account for our analysis.

Precipitation data are from Climatic Research Unit time-series data sets (CRU TS3.21) at 0.5° resolution. An additional air temperature data set—the Global Historical Climatology Network-Monthly, which is used in the analysis of LST versus air temperature—can be obtained from http://www.ncdc.noaa.gov/ghcnm/v3.php. We use the homogeneity adjusted temperature records for our analysis. CRU precipitation data and Global Historical Climatology Network temperature data are from January 2002 to December 2012.

Digital elevation model is the 1-km Shuttle Radar Topography Mission 30 (SRTM30) version 2.1, which is resampled into 0.05° to match MODIS data.

All MODIS variables in the analysis, except land cover, are based on multi-year averages between 2002 and 2013, which are first calculated at the MODIS data composite time scale of each variable (for example, 8 days for LST or 16 days for albedo) and then aggregated to monthly, seasonal, and annual means. MODIS data, including LST, albedo, ET, and land cover, have the spatial resolution at Climate Modeling Grid (0.05°). Extracting high-quality satellite data only (selected by MODIS QC) inevitably results in some data gaps. For example, there are less valid albedo data in tropical and high-latitude regions primarily due to the unfavourable atmospheric conditions like cloud contamination^49^ and high solar zenith angles^50^. Our analysis is based on the climatology values in the period 2002–2013 to increase the number of available high-quality data and to minimize the influence of interannual climate variability.

### Window searching strategy

We apply a window searching strategy to find all available samples to compare forest with open land across the globe (Supplementary Fig. 1). This strategy ensures that all forests and open lands that are close in distance and share similar climate background are compared. Forests near grass/crop lands are also considered to be most susceptible to future land cover change. The search window size is 9 × 5 pixels (longitude × latitude), approximately equal to 50 km × 28 km. Two adjacent windows are partially overlapping along both the longitudinal (4 pixels) and latitudinal (2 pixels) directions. If both forest and open land pixels exist within a window, it would be a valid comparison sample and subsequently we calculate the mean differences in LST, ET, albedo, elevation, and so on, of forest minus open land. Snow frequency of each sample is the average of all forest and open land pixels within a window, and precipitation is taken from the coarser overlapping CRU precipitation grid.

### Elevation adjustment

Because forest and open land pixels may have different elevations even within one window, a systematic bias appears in ΔLST due to lapse rate. To correct this bias, we perform an elevation adjustment by subtracting the elevation-induced ΔLST from the original value (Supplementary Fig. 4). This correction term is the product of the elevation difference (ΔELV) and the regression slope (*b*) derived from linear regression of ΔLST versus ΔELV. Hence,


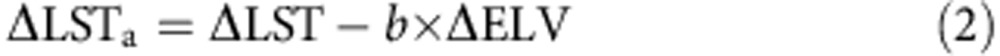


where ΔLST_a_ is the adjusted LST difference. To avoid artificial signals resulting from the adjustment, especially when the elevation difference is very large, we constrain our analysis to samples whose elevation differences are within ±500 m. After elevation adjustment, we obtain 11,530 valid comparison samples in the analysis.

### LST versus air temperature

Air temperature is recognized to be generally dependent on LST^51^. They are closely coupled, but coupling strength varies across different land covers 52 and weather conditions (cloudy sky or clear sky)^51^. In addition, LST and near-surface air temperature have different physical meanings and influencing factors^39^, thus temperature effects based on LST could be different from the effects based on air temperature. Taking these differences into consideration can avoid a possible misinterpretation that could arise from the use of different temperature metrics^14^.

To better understand this issue, we attempted to estimate the possible influence of quantifying temperature effects using LST versus air temperature.

We compared MODIS LST and air temperature at all available weather stations of the Global Historical Climatology Network-Monthly during the study period that are classified as either forest or open land (312 for forest and 944 for open land) by MODIS land cover data. Coupling between LST and air temperature is stronger in forest than in open land, as indicated by the higher correlation coefficient in forest (*r*=0.87 and 0.83 for maximum and minimum temperature, respectively) than in open land (*r*=0.83 and 0.76 for maximum and minimum temperature, respectively). We define a term *δ* as the difference between LST and air temperature:


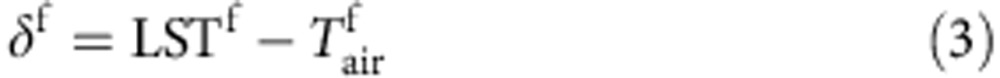



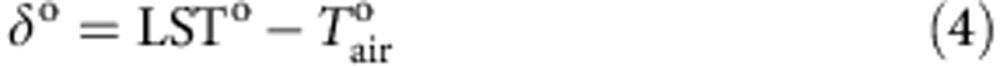


where *T*_air_, LST, and *δ* represent air temperature, LST, and their difference, respectively, and superscripts f and o denote forest and open land.

Supplementary Fig. 5 shows the LST-air difference term *δ* in maximum temperature and minimum temperature as a function of latitude (averaged to 1° latitude band). Positive *δ* means LST is higher than air temperature and vice versa. The difference term *δ* is positive in maximum temperature for open land at low and mid latitude but negative at high latitude (Supplementary Fig. 5a). For forests, it is negative at almost all latitudes (Supplementary Fig. 5c). As for minimum temperature, a negative *δ* is found in both open land (Supplementary Fig. 5b) and forests (Supplementary Fig. 5d) at nearly all latitudes. This confirms that there is a systematic difference between LST and air temperature across latitudes as indicated by a previous study^52^.

Using latitude LST-air difference term *δ* (equations (3) and (4), and panels (e) and (f) in Supplementary Fig. 5), we can infer possible influence of temperature metrics on the ΔLST latitudinal pattern by assuming such a difference applies to all forests and open lands within a given latitude range (equation (5)).


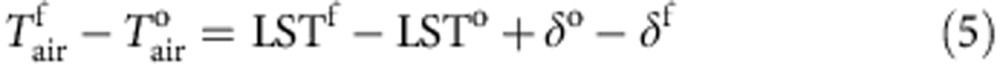


The overall effect of the correction term (*δ*^o^−*δ*^f^) is to dampen the magnitude of the original ΔLST (LST^f^−LST^o^) pattern for both maximum and minimum temperature (Supplementary Fig. 5e,f). Another study^43^ has shown that land use change has a smaller effect on air temperature than on LST. For instance, the daytime warming in boreal forests and cooling in tropical forests that come from LST would be reduced in air temperature and there would be less night warming in mid latitudes. Similarly, it can be inferred that the daily air temperature difference between forest and open land should be smaller than the daily ΔLST. This is because LST is more sensitive to land surface characteristics and surface energy budget^53^ and has larger diurnal variations than air temperature. However, such qualitative analysis is unable to give an accurate estimate for air temperature because the bias and correction term are estimated at the locations of the Global Historical Climatology Network stations rather than the actual locations of each comparison sample. So the correction term cannot be directly applied to the ΔLST pattern to get the ‘inferred’ or ‘pseudo’ air temperature difference.

Another source that may contribute to the difference term, *δ,* is using only clear-sky LST data to calculate ΔLST; limited *in situ* surface temperature measurements do not allow us to perform a similar analysis as shown above. Nevertheless, some insights can be gained from the existing literature. A study that compared *in situ* LST and air temperature found that the difference between LST and air temperature is much smaller under cloudy sky than under clear sky^51^. This means that cloudy-sky LST is similar to air temperature. With analogy between LST and air temperature, as they are very close under cloudy sky, we tentatively infer that ΔLST under cloudy sky should be smaller than ΔLST observed under clear sky. Therefore, it is expected that ΔLST under all sky conditions should be smaller than the results presented in this paper, which is for clear-sky only. However, this inference has not been independently verified with data and thus should be treated with caution.

### Sensitivity test

We perform sensitivity tests for the definition threshold of forest and open land (70, 60 and 50% areal coverage), size of search window (9 × 1, 5 × 3 and 5 × 1), and elevation adjustment (±100 m, no adjustment). Our results are robust against these choices of parameters.

Different thresholds of 70, 60 and 50% for defining forest and open land based on their areal percentages are tested (Supplementary Fig. 6). The latitude patterns are consistent through different thresholds. A higher threshold generates a slightly larger ΔLST because a denser forest has a more evident impact. The magnitude of local cooling of a forest also depends on its coverage proportion^32^.

Different search windows at sizes of 9 × 1, 5 × 3 and 5 × 1 are tested (Supplementary Fig. 7). A smaller search window yields fewer comparison samples and thus higher uncertainty at some latitudes. The identified pattern is not significantly affected by the choice of the window size.

We compare the results with more stringent elevation control (±100 m) as well as no elevation adjustment but the elevation difference is set to be within ±500 m (Supplementary Fig. 8). The more stringent elevation control yields fewer comparison samples and larger confidence interval , but the identified LST pattern is essentially the same. Without elevation adjustment, we see a similar pattern with slight differences at some latitudes where elevation difference is larger. Elevation adjustment has less influence on the results when the elevation control is more stringent. Therefore, our results are unlikely to be an artificial effect due to elevation adjustment.

## Author contributions

Y.L. designed the research. Y.L. performed the research and analysed the data. M.Z. and Q.M. developed the evapotranspiration data. Y.L., S.M., M.Z., and E.K. drafted the manuscript. All authors contributed in the discussion of the results and in writing the paper.

## Additional information

**How to cite this article:** Li, Y. *et al.* Local cooling and warming effects of forests based on satellite observations. *Nat. Commun.* 6:6603 doi: 10.1038/ncomms7603 (2015).

## Supplementary Material

Supplementary Information Supplementary Figures 1-8 and Supplementary Tables 1-4
