# Supplementary material for: Local cooling and warming effects of forests based on satellite observations
Source: Nat Commun. 2015 Mar 31;6:6603. doi: 10.1038/ncomms7603 (PMC4389237; doi:10.1038/ncomms7603)
Supplement: Supplementary Information — Supplementary Figures 1-8 and Supplementary Tables 1-4 [file ncomms7603-s1.pdf]

Supplementary information:

Supplementary figures

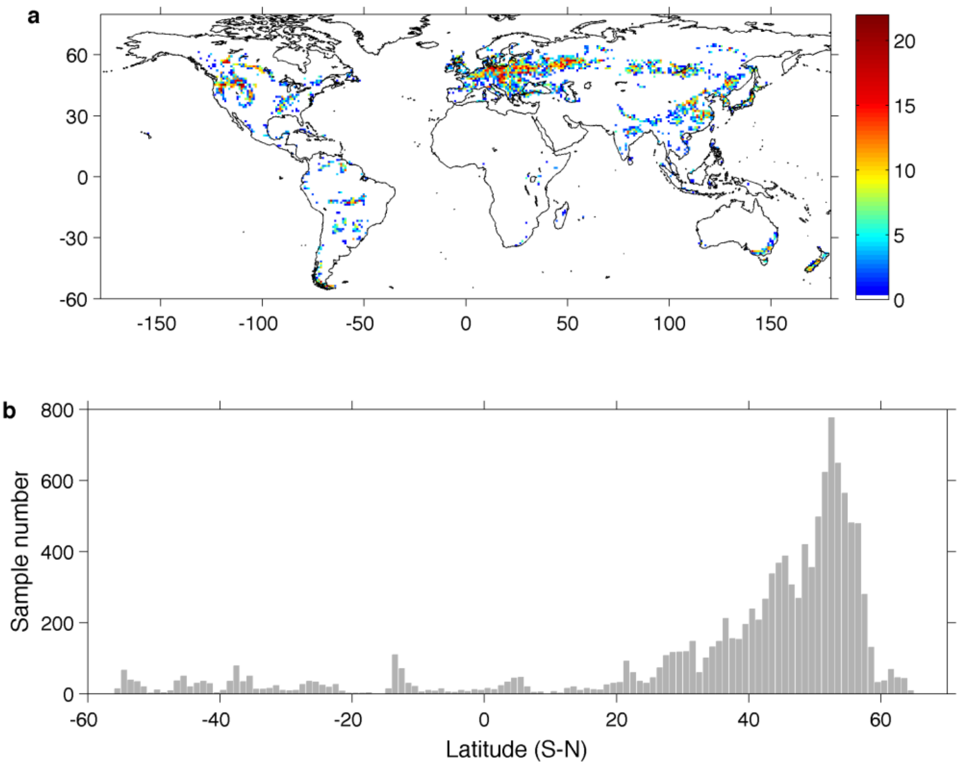

Supplementary Figure 1. Geographical and latitudinal distribution of comparison samples between forest and open land. **(a)** Sample number aggregated on 1×1 degree grids. **(b)** Sample number aggregated at each one degree latitude.

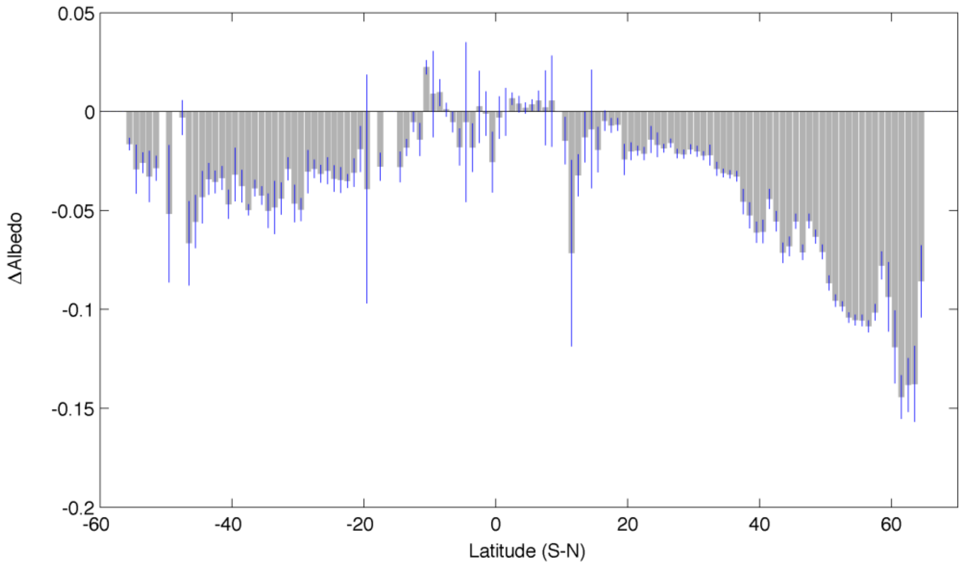

Supplementary Figure 2. Latitudinal albedo difference of forest minus open land based on GLASS data. Latitude bars with CI out of display range are not shown.

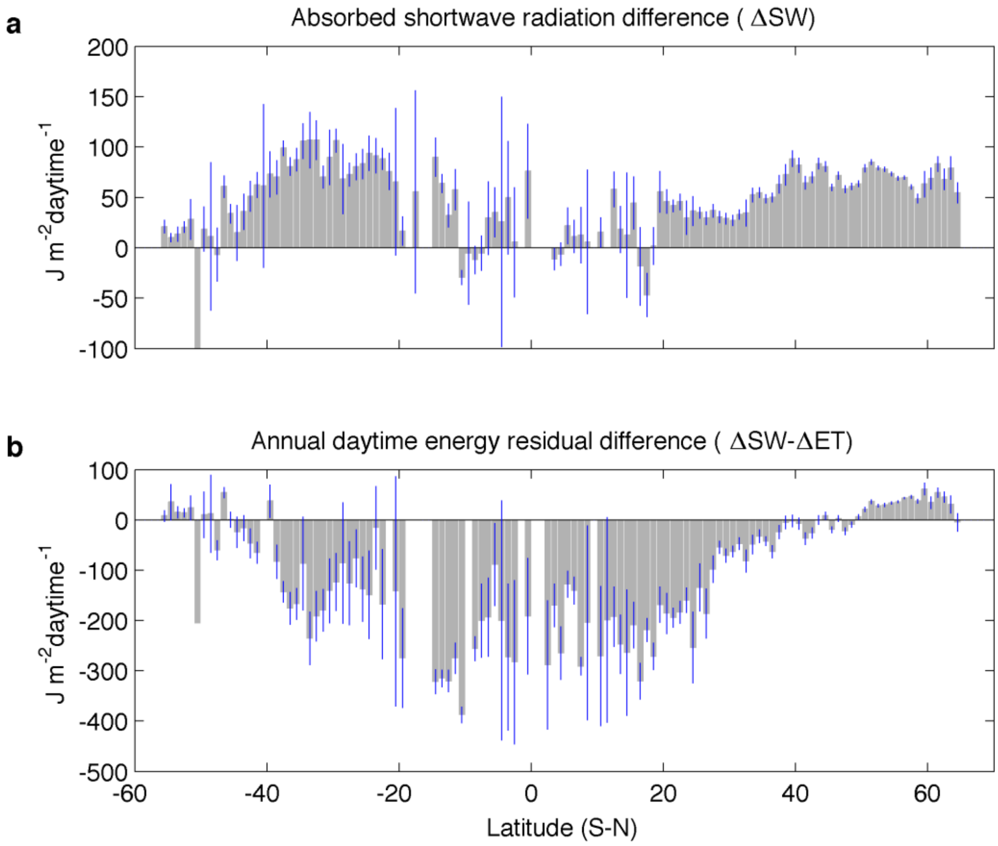

Supplementary Figure 3. Energy balance of forest minus open land. **(a)** Annual absorbed shortwave radiation difference of forest minus open land (ΔSW). A positive value means that forests absorb more energy than open land during the day. Such a latitude pattern with a more positive value in mid latitude is a combined result of declining shortwave radiation (SW) and increasing albedo difference (Δalbedo) from the tropics to high-latitude, since ΔSW=Δalbedo ×SW. **(b)** Annual daytime energy residual of forest minus open land (ΔSW-ΔET), reflecting how much absorbed energy is offset by ET (latent heat). The remaining energy warms the surface. A positive value means that forests have a larger energy surplus for heating the surface. The blue line denotes 95% CI estimated by *t*-test. Latitude bars with CI out of display range are not shown.

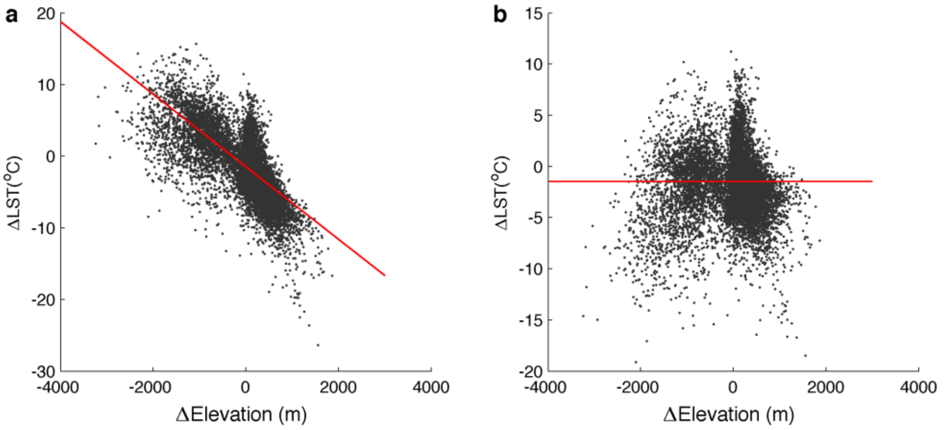

Supplementary Figure 4. LST difference of forest minus open land before **(a)** and after **(b)** elevation adjustment. The data shown are annual daytime ΔLST and their elevation difference.

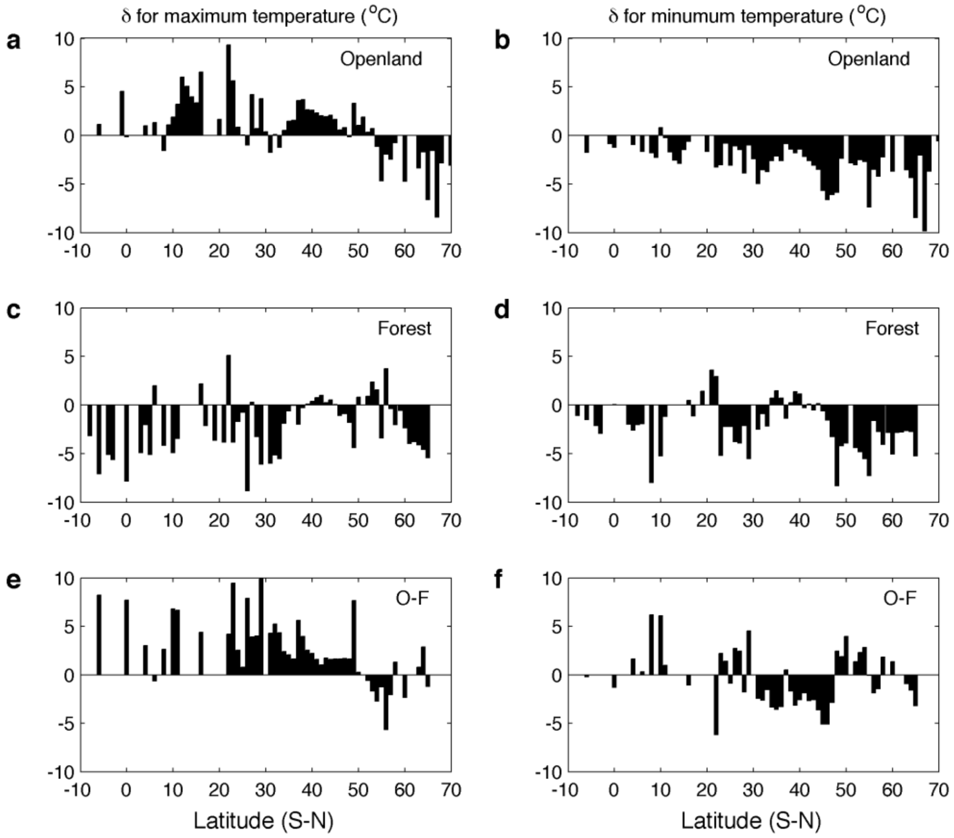

Supplementary Figure 5. Latitudinal difference of LST minus air temperature in forest and open land. Positive δ means LST is higher than air temperature, and vice versa. Left panel shows δ for maximum temperature, and right panel shows δ for minimum temperature. **(e)** and **(f)** show correction term δ^{o}-δ^{f}, which can be used to infer the influence of temperature metrics on latitudinal temperature pattern.

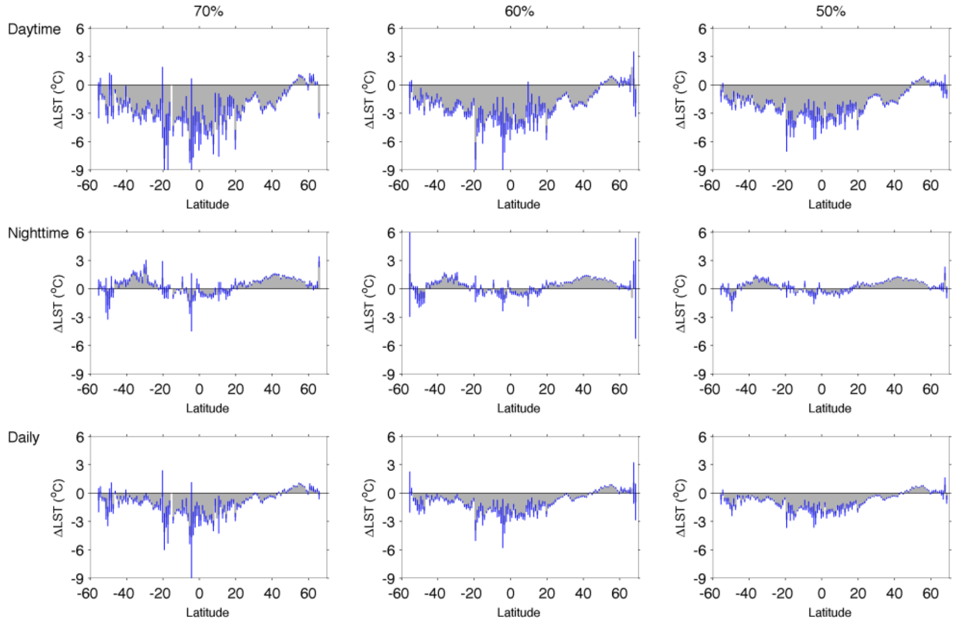

Supplementary Figure 6. Latitude pattern of LST difference of forest minus open land with different thresholds for defining forest and open land (70%, 60%, and 50%). Unlike other latitudinal figures in main text and supplementary information, CI here is drawn for every latitude bar even if it exceeds the display range. This also applies to Supplementary Figs. 7 and 8 for the purpose of sensitivity tests.

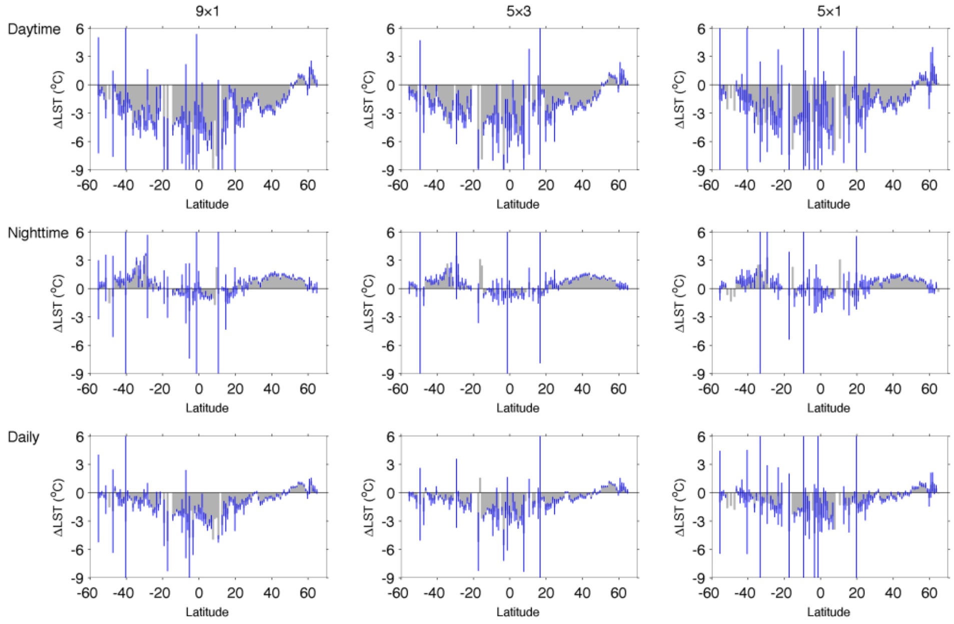

Supplementary Figure 7. Latitude pattern of LST difference of forest minus open land with different window sizes (9 × 1, 5 × 3, and 5 × 1).

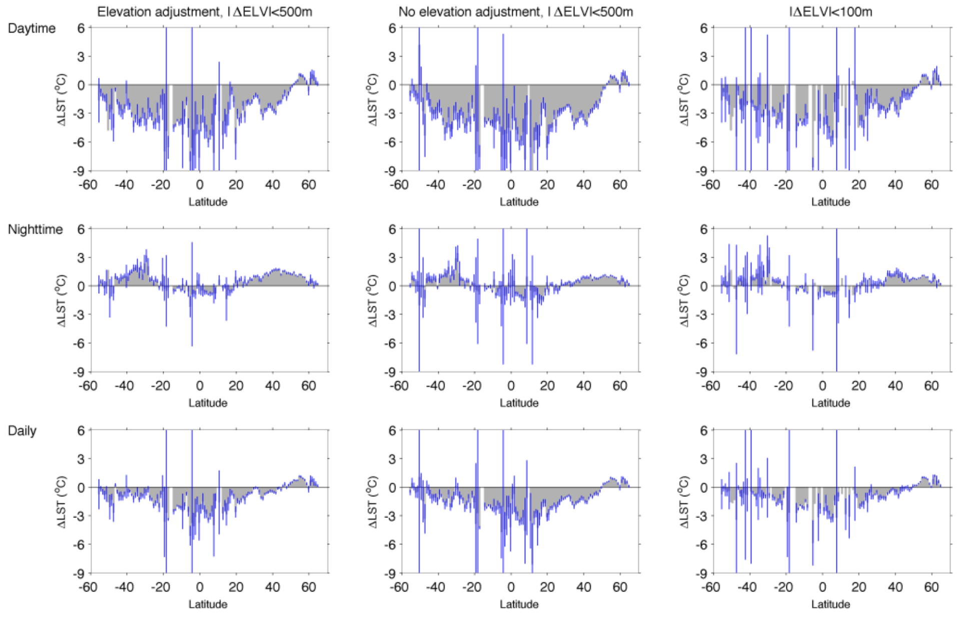

Supplementary Figure 8. Latitude pattern of LST difference of forest minus open land with different elevation controls (elevation adjustment with ΔELV < ±500m, no elevation adjustment but ΔELV < ± 500m, and ΔELV < ±100m).

Supplementary Tables

Supplementary Table 1. Annual LST difference of forest minus open land. (°C)

	Day	Night	Daily
>50°N	0.51±0.05	1.08±0.03	0.79±0.03
20°N-50°N	-1.82±0.06	1.29±0.03	-0.27±0.03
20°S-20°N	-4.40±0.17	-0.42±0.06	-2.41±0.10
20°S-50°S	-3.02±0.12	1.08±0.08	-0.97±0.07
>50°S	-1.25±0.23	0.26±0.25	-0.50±0.19

Mean ± 95% CI estimated by *t*-test. CI reflects the spatial variability of the mean within a geographical zone (same as Supplementary Table 1 to 4).

Supplementary Table 2. Seasonal LST difference of forest minus open land. (°C)

	DJF			MAM			JJA			SON		
	Day	Night	Daily	Day	Night	Daily	Day	Night	Daily	Day	Night	Daily
>50°N	2.76 ±0.08	1.65 ±0.06	2.20 ±0.06	-0.37 ±0.05	1.16 ±0.03	0.40 ±0.03	-2.98 ±0.05	0.70 ±0.02	-1.14 ±0.02	-0.27 ±0.04	0.81 ±0.03	0.27 ±0.02
20°N-50°N	0.29 ±0.08	1.60 ±0.04	0.94 ±0.05	-2.46 ±0.06	1.30 ±0.03	-0.58 ±0.04	-3.67 ±0.07	0.80 ±0.03	-1.44 ±0.04	-2.11 ±0.04	1.46 ±0.03	-0.32 ±0.02
20°N-20°S	-3.87 ±0.27	-0.41 ±0.09	-2.13 ±0.13	-4.03 ±0.23	-0.55 ±0.08	-2.29 ±0.12	-4.80 ±0.21	-0.32 ±0.08	-2.54 ±0.11	-4.90 ±0.19	-0.41 ±0.07	-2.65 ±0.11
20°S-50°S	-4.69 ±0.20	0.84 ±0.09	-1.92 ±0.10	-2.71 ±0.13	1.25 ±0.09	-0.73 ±0.08	-1.41 ±0.11	1.25 ±0.09	-0.08 ±0.07	-3.22 ±0.16	1.00 ±0.09	-1.11 ±0.09
>50°S	-2.54 ±0.27	0.22 ±0.34	-1.20 ±0.20	-1.12 ±0.22	0.18 ±0.28	-0.46 ±0.21	0.71 ±0.33	0.56 ±0.22	0.64 ±0.23	-2.33 ±0.27	0.10 ±0.27	-1.12 ±0.19

Mean ± 95% CI estimated by *t*-test. DJF (December-January-February), MAM (March-April-May), JJA (June-July-August), SON (September-October-November). Definitions for season are the same throughout Supplementary Table 2 to 4.

Supplementary Table 3. Annual and seasonal albedo differences of forests minus open land.

	Annual	DJF	MAM	JJA	SON
>50°N	-0.12±0.001	-0.26±0.003	-0.11±0.002	-0.03±0.000	-0.08±0.001
20°N-50°N	-0.06±0.001	-0.12±0.003	-0.04±0.001	-0.03±0.000	-0.04±0.000
20°S-20°N	-0.02±0.002	-0.02±0.002	-0.02±0.002	-0.02±0.002	-0.01±0.001
20°S-50°S	-0.05±0.003	-0.05±0.002	-0.05±0.001	-0.06±0.004	-0.05±0.003
>50°S	-0.03±0.003	-0.03±0.002	-0.03±0.002	-0.04±0.001	-0.03±0.002

Mean ± 95% CI estimated by *t*-test.

Supplementary Table 4. Annual and seasonal ET differences of forest minus open land as well as annual background precipitation and shortwave radiation. ET here is daily total. (mm yr^{-1} for ET and precipitation, J m^{-2} $daytime^{-1}$ for shortwave radiation)

	Annual	DJF	MAM	JJA	SON	Precipitation	Shortwave radiation
>50°N	49±1.6	-1± 0.1	9± 0.4	36± 1.2	5± 0.3	607±7	1006±3
20°N-50°N	141± 4.0	4± 1.0	31± 1.2	72± 1.6	33± 1.1	827±10	1418±4
20°S-20°N	428± 14.6	91± 4.1	95 ±4.6	120± 5.5	122± 5.2	1703±44	1714±10
20°S-50°S	238± 16.6	111± 7.1	51± 4.1	17± 7.1	60± 5.5	1051±41	1608±17
>50°S	6±38.8	15± 12.4	-3± 9.6	-7± 7.7	1± 9.9	522±13	915±6

Mean ± 95% CI estimated by *t*-test.
